# Correction to “Development of a Scale to Assess the Safe Disposal of Insulin Pen Needles in Patients With Diabetes: A Delphi Study”

**DOI:** 10.1002/nop2.70553

**Published:** 2026-06-15

Zhang, W., J. Ge, Y. Han, L. Zheng, R. Li, and W. Yang. 2026. “Development of a Scale to Assess the Safe Disposal of Insulin Pen Needles in Patients With Diabetes: A Delphi Study.” *Nursing Open* 13, no. 2: e70394. https://doi.org/10.1002/nop2.70394.

In the above mentioned article, there was an error in the author affiliations. Ruihong Li was listed as being affiliated with two institutions. However, Ruihong Li is currently affiliated with only one institution. The correct affiliation is as follows:

Shanghai Xuhui District Central Hospital, Shanghai, China.

Additionally, there is a data transcription error in the published article. In Table 2, the value of the Kendall's coefficient of concordance for the second round is listed as “0.180.” This should be listed as “0.200.”

We apologize for these errors.

